# Effect of diffusion kinetics on the ice nucleation temperature distribution

**DOI:** 10.1038/s41598-022-20797-1

**Published:** 2022-09-29

**Authors:** Lorenzo Stratta, Andrea Arsiccio, Roberto Pisano

**Affiliations:** Molecular Engineering Laboratory (molE), Department of Applied Science and Technology, Politecnico di Torino, 24 corso Duca degli Abruzzi, 10129 Turin, Italy

**Keywords:** Chemical engineering, Chemical physics, Macromolecules and clusters

## Abstract

The nucleation behavior of water is crucial in many fields, spanning meteorology, glaciology, biology, and astrophysics. We report observations suggesting an effect of diffusion kinetics in water on the heterogeneous immersion/contact mode nucleation temperature distribution of ice. We performed differential scanning calorimetry analyses of repeated freeze/thaw cycles and investigated the effect of several variables on the regularity of the nucleation temperature distributions obtained. We observed that the thawing temperature and residence time above 0 °C affect the width of the measured distributions. We explain the observed phenomena according to the diffusion behavior of an external nucleator. Specifically, conditions of enhanced diffusion of the nucleator translated into broader, more scattered distributions, while conditions of limited diffusion translated into narrower, more regular distributions. Lastly, based on our experimental findings, we propose a theoretical explanation centered on the temperature dependence of diffusion kinetics in water.

## Introduction

In the past decades, the determination of the ice nucleation temperature (*T*_*n*_) has been crucial in many technological fields. Heterogeneous nucleation data are used, for example, in meteorology^1–3^ to predict the formation of ice in the clouds, in aerospace applications^4,5^ to avoid icing phenomena, in pharmaceutics^6^ to control product quality and process efficiency, and in biology^7,8^ to understand how certain animals can withstand hibernation without critical damage to cells and tissues or protein degradation^9^.

The nucleation of water is a complex phenomenon characterized by an energy barrier for the formation of a stable nucleus, and a metastable zone, where no appreciable nucleation occurs despite being beyond the thermodynamic equilibrium point. Both homogeneous nucleation, i.e., nuclei formation in the bulk solution, and heterogeneous nucleation, i.e., the generation of nuclei onto external surfaces or foreign bodies, may occur^10,11^. However, the exceptionally high energy barrier associated with homogeneous nucleation makes it hard to occur spontaneously until very low temperatures are reached (− 40 °C or even below^12–16^). Ice nucleation is generally heterogeneous, at least in a typical experimental setup, as the energy barrier is lowered by the presence of foreign particles^17–19^.

In the last few years, new insights into the homogeneous and heterogeneous nucleation of water have been provided by applying molecular dynamics simulations that can accurately describe the molecular level phenomena involved^20,21^. However, even if promising, molecular dynamic simulations are limited by their high computational cost to short time scales and cannot deal with too big, complex systems.

Previous studies in the field investigated the nucleation process, analyzing the effect of several variables such as cooling rate^22,23^, sample volume^24,25^, presence of nucleating agents^23,26,27^, and different materials of the support^28,29^, primarily focusing on the effect of the latter on the average nucleation temperature. However, both the holding time between subsequent cycles and the temperature reached by the melt were neglected, most of the time not even reported, due to the assumption that the solution would equilibrate instantaneously once the ice was completely melted due to the short lifetime of hydrogen bonds^30,31^. In 2008, Vali^26^ reported a possible effect of the time spent in the melt on the nucleation temperature distribution, when a few repeated freeze/thaw experiments had to be stopped overnight and the distributions of the nucleation temperatures showed some anomalies as a consequence. Vali suggested that these changes in nucleation temperature could “*arise from alterations of the nuclei, some being permanent and some transitory*”, but also specified that “*the experiments provide only a diagnosis of these alterations and interpretation of what causes them can only be speculative*”^26^.

In this work, we extend the investigation of the effect of external variables on the nucleation behavior of ice. For this purpose, we performed repeated freeze/thaw experiments on pure water using differential scanning calorimetry (DSC), and concomitantly measured the nucleation temperature. These experiments produced dispersed distributions that we then plotted as survival curves (i.e., cumulative distributions of samples with a nucleation temperature above a given value), which provided information about the average nucleation temperature and its inherent variability. The effect of several parameters was investigated during these DSC runs, including the cooling/heating rate, the thawing and freezing temperatures, the holding time at various thawing temperatures, and the sample mass. In line with Vali’s considerations, our results indicate that both the temperature reached by the melt between subsequent cycles and the holding time at that temperature directly affects the nucleation temperature distribution of ice. We propose an explanation for these observations based on the temperature and time-dependence of diffusive processes in water.

## Results

Several DSC experiments were performed, as detailed in the “Materials and methods” section and Table 1. The nucleation temperature was extracted from the DSC data, and the obtained distributions were converted to survival curves. It is important to emphasize that DSC experiments differ from micro-volume experiments, where the survival curve of many droplets/wells of the same water can be measured in one cooling cycle. In our case, survival curves were instead collected through multiple heating/cooling cycles performed by the DSC. The temperature at which the survival curves reach the value of 0.5 (*T*_*50*_) is defined as the median nucleation temperature of the system, while the difference between the temperatures crossing the 0.1 (*T*_*10*_) and 0.9 (*T*_*90*_) lines can be defined as the width of the distribution (*T*_10_-*T*_90_). The median and the average of the distribution could be equal if the distribution is symmetric, but this condition is seldom verified. Another parameter giving information about the dispersity of a distribution is the variance, defined as the sum of the square deviations from the average. Having used the median temperature, instead of the average, to characterize the distribution, we defined the variability of the distribution (*V*_50_) as the sum of the square deviations from the median (*T*_50_).

**Table 1:** Conditions used for the DSC experiments.

Run	*m*_w_, mg	*R*, °C/min	*T*_th_, °C	*T*_f_, °C	*t*_h_, min	*τ*, min	*n*_c_, –	*T*_50_, °C	*T*_10_*–T*_90_, °C	*V*_50_, °C^2^
I	16.5	5	20	− 40	1	9	99	− 22.5	0.8	0.29
II	16.6	7.5	30	− 30	1	9	100	− 21.5	1.2	0.38
III	17.0	10	40	− 40	1	9	100	− 24.0	1.7	0.55
IV	16.9	12.5	50	− 40	1	9	100	− 20.2	3.8	1.90
V	16.7	15	60	− 40	1	9	100	− 21.8	5.7	4.87
VI	15.4	15	60	− 40	1	9	41	− 25.0	4.6	3.47
		10	40			9	41	− 23.3	2.2	0.72
		5	20			9	41	− 22.0	2.0	0.71
VII	17.5	5	20	− 40	1	9	41	− 20.9	2.8	1.30
		10	40			9	41	− 23.2	2.2	0.55
		15	60			9	41	− 22.6	4.9	4.47
VIII	15.3	15/10	60	− 40	1	9	40	− 23.4	5.2	4.82
		10/10	40			9	40	− 25.6	1.8	0.64
		5/10	20			9	40	− 25.4	2.0	0.42
IX	15.2	5/10	20	− 40	1	9	40	− 24.6	1.2	0.16
		10/10	40			9	40	− 24.5	0.7	0.07
		15/10	60			9	40	− 24.5	4.6	2.34
X	12.0	5	20	− 40	60	68	99	− 20.2	6.5	7.85
XI	10.5	10	20	− 40	1	5	99	− 17.8	1.9	1.23
XII	10.2	10	20	− 40	60	64	100	− 17.2	4.8	3.42
XIII	17.1	5	20	− 40	5	13	100	− 22.5	3.9	2.15
XIV	17.1	5	20	− 40	15	23	100	− 25.8	8.1	9.30
XV	16.1	5	20	− 40	30	38	100	− 22.4	0.7	0.11
XVI	17.1	7.5	20	− 40	1	6.3	100	− 23.2	5.3	7.38
XVII	16.3	10	20	− 40	1	5	100	− 23.7	0.8	0.10
XVIII	17.1	12.5	20	− 40	1	4.2	50	− 20.6	1.0	0.18
XIX	16.0	15	20	− 40	1	3.7	100	− 25.8	2.4	0.77
XX	17.0	17.5	20	− 40	1	3.3	100	− 18.2	3.2	1.71
XXI	16.2	20	20	− 40	1	3	100	− 21.2	1.4	0.42
XXII	12.6	10	20	− 40	1	5	100	− 24.3	2.0	1.11
XXIII	16.0	10	20	− 40	1	5	100	− 24.1	2.3	0.77
XXIV	15.1	8.3	20	− 80	1	5.8	94	− 22.5	1.1	0.95

In each DSC experiment, we performed 40 < *n*_*c*_ < 130 cycles between two temperature values (freezing final value *T*_*f*_ and thawing final value *T*_th_), at selected cooling/heating rates *R* (Table 1). The DSC pans were held at the thawing final temperature *T*_th_ for a given time *t*_h_ to ensure complete thawing.

**Figure 1:**
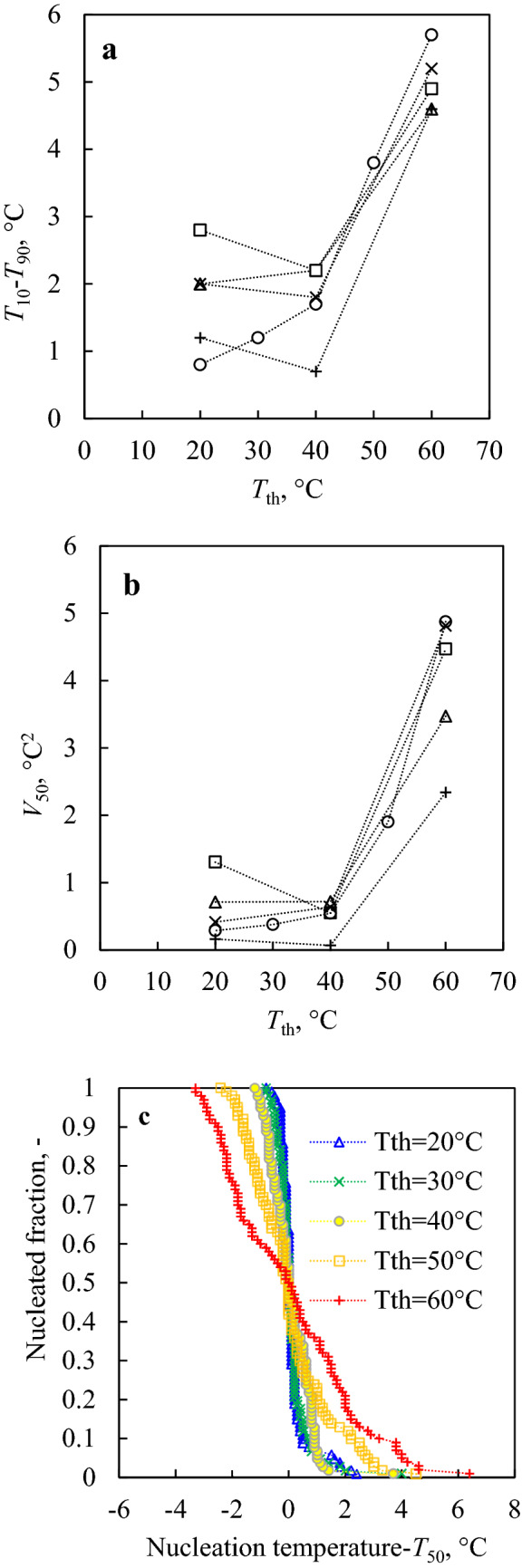
Width of the nucleation temperature distribution (**a**: *T*_10_-*T*_90_, in °C, **b**: *V*_50_, in °C^2^) versus the maximum thawing temperature *T*_th_. All the experiments shown in this figure (I–IX in Table 1) are characterized by *τ* = 9 min. The width of the *T*_*n*_ distributions increases as *T*_th_ increases from 20 to 60 °C. In (**a**) and (**b**): circles correspond to experiments I–V, triangles to experiment VI, squares to experiment VII, crosses to experiment VIII, and pluses to experiment IX. **c**: Nucleated fraction versus nucleation temperature translated by *T*_50_ (experiments I–V in Table 1). As the maximum thawing temperature rises from 20 to 60 °C, the survival curves stretch over the horizontal axis, indicating a broader nucleation temperature distribution.

We first asked ourselves whether different values of *T*_th undefined_ could affect the stochasticity of the nucleation temperature distributions. As the diffusive processes in water strongly depend on temperature, higher values of *T*_th_ correspond to conditions of higher thermal agitation of the molecules in the melt. We, therefore, conducted a set of experiments (I–IX in Table 1), shown in Fig. 1, at different values of *T*_th_ ranging from 20 to 60 °C. All the experiments were performed with a constant residence time *τ* of the sample at temperatures above 0 °C (*τ* = *t*_h_ + 2*T*_th_/*R*, where *T*_th_ is in °C) equal to 9 min. In experiments I-V each sample was cycled between the same temperatures *T*_*f*_ and *T*_th_ with constant *R* and *t*_h_. The water sample and DSC pan were changed between each of these first five experiments. On the contrary, in experiments VI-IX, the same water sample was subjected to three subsequent sub-sets of conditions, with decreasing (VI, VIII) or increasing (VII, IX) values of *T*_th_ (as detailed in Table 1). These tests aimed to study the behavior of the same system under different sets of conditions, thus excluding the possibility of false-positive correlations caused by random differences (e.g., presence of different external nucleators) between different DSC pan/water droplet systems. In experiments VI and VII the cooling rate in each sub-set was kept constant along the whole temperature ramp. However, even though this procedure guarantees a constant *τ* of 9 min, different values of *R* correspond to different residence times in the supercooled state, possibly influencing the median nucleation temperature of each sub-set. In order to exclude this effect from the analysis, experiments VIII and IX were performed using two different *R* values for each sub-set. Specifically, for sample temperatures above 0 °C *R* was varied between the sub-sets so as to maintain in all cases a constant *τ* of 9 min, while *R* = 10 °C/min was used below 0 °C for all the sub-sets of conditions.

As *T*_th_ was systematically increased from 20 to 60 °C, both *T*_10_-*T*_90_ and *V*_50_ grew (Fig. 1a,b). This effect is visible in Fig. 1c, where the survival curves, horizontally shifted by their respective *T*_50_, are broader as *T*_th_ increases. Experiments VI-IX confirm this trend and suggest a direct correlation between the nucleation temperature distribution and the thermal agitation of the molecules in the melt between subsequent cycles. Even though it is true that some experiments show some decreasing tendency between 20 and 40 °C, this trend is not valid for all the experiments. On the other hand, the increasing tendency shown between 40 and 60 °C is consistent in all the experiments. We believe that the effect seen between 20 and 40 °C manifests the stochastic nature of ice nucleation for distributions that are very narrow overall.

**Figure 2:**
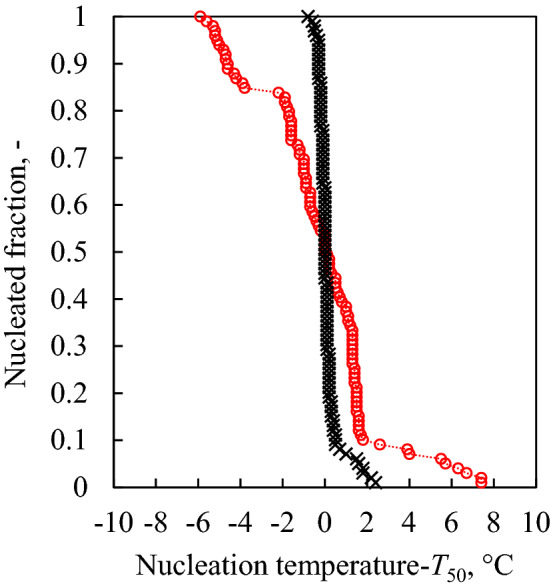
Nucleated fraction versus nucleation temperature translated by *T*_50_ (experiments I and X in Table 1). When *τ* = 9 min (black crosses, test I in Table 1), the distribution is narrow while it becomes wider when *τ* = *68* min (red circles, test X in Table 1).

Therefore, we tested whether other conditions of high thermal agitation could influence the nucleation temperature distribution similarly to *T*_th_. For this purpose, experiment X was conducted with a *t*_h_ of 60 min. Our working hypothesis was that if the thermally-enhanced diffusion in water influences the nucleation temperature distribution, time-enhanced diffusion could have the same effect. Indeed, we observed that the nucleation temperature distribution seems to be strongly correlated to *τ* as well, as shown in Fig. 2. Specifically, low values of *τ* were associated with a uniform and sharp distribution of nucleation temperatures (black curve), while high values of *τ* corresponded to highly dispersed nucleation temperatures (red curve). Supplementary Fig. S1 shows the whole thermogram collected during run X. Such a thermogram shows that the nucleation temperatures are widely scattered, while the melting peaks are superimposed for all the heating/cooling cycles. This result confirms that the scatter observed in the nucleation temperature distributions is not an artifact of the instrument (as, in this case, the melting temperatures would have been scattered as well) but rather an actual property of the nucleation behavior of water.

**Figure 3:**
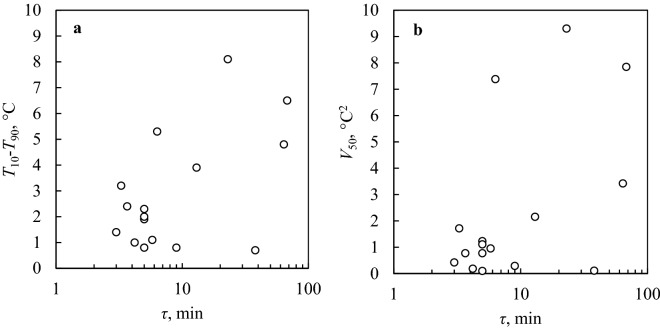
Width of the nucleation temperature distribution (**a**: *T*_10_-*T*_90_, in °C, **b**: *V*_50_, in °C^2^) versus the residence time above 0 °C (*τ*, in logarithmic scale). All these experiments were performed at *T*_th_ = 20 °C (numbers X–XXIV in Table 1). The omnibus test suggests the presence of a significant correlation between *τ* and both *T*_10_-*T*_90_ (F-value 14.4, degrees of freedom 12, p-value 0.02) and *V*_50_ (F-value 44.4, degrees of freedom 12, p-value 0.005). The number of degrees of freedom represents the number of independent subgroups in the considered dataset. The F-value is calculated by dividing the variance of the subgroups’ means by the mean of the within-subgroup variances. The p-value, instead, indicates the probability of obtaining results at least as extreme as those observed without any correlation between the input variables (in this case, *τ* and *T*_10_-*T*_90_ or *τ* and *V*_50_). Large F-values and small p-values are indicative of the correlation.

We further investigated this phenomenon by collecting the results of a set of experiments (X-XXIV in Table 1) at *T*_th_ = 20 °C. As can be seen in Fig. 3, there is an increase in *T*_10_–*T*_90_ value as a function of *τ*, with *T*_10_–*T*_90_ eventually rising to 8 °C in the case of a high value of *τ*. Furthermore, a statistically relevant correlation was observed between the values of *τ*, *T*_10_-*T*_90_, and *V*_50_. As a control, experiments XI, XVII, XXII, and XXIII were conducted with the same procedure (*T*_th_ = 20 °C, *R* = 10 °C/min, and *τ* = 5 min), giving in all cases values of *T*_10_-*T*_90_ lower than 2.5 °C. In contrast, we verified that *τ* has no statistically relevant effect on the median nucleation temperature *T*_50_ (Supplementary Fig. S2).

In order to thoroughly examine the effect of different process variables on the nucleation temperature distribution, we carried out further experiments. We first verified whether the size of ice crystals formed during freezing might somehow affect the distribution of nucleation temperatures. It is well-known in the literature that the ice crystal size is linked to both nucleation temperature and cooling rate^32^. Therefore, we first evaluated any direct correlation between *T*_50_ and the *T*_n_ distribution (Supplementary Fig. S3), and no statistical correlation was observed. This result is essential as it allows the comparison between distributions centered at different *T*_50_. Furthermore, the possible effect of *R* was further examined by performing a series of experiments (I and XVI-XXI in Table 1) with constant *T*_th_ = 20 °C and *τ* shorter than 10 min. These experiments showed no statistical correlation between *R* and neither *T*_50_ nor *T*_10_-*T*_90_ (Supplementary Fig. S4). As anticipated, one would expect different R values to influence *T*_50_ as they produce different residence times in the supercooled state. However, this would be true only if the type of nucleator remained unaltered between the different experiments. Given the heterogeneous nature of nucleation observed in this study and that each of the experiments above was conducted on a different DSC pan/water sample combination, with a consequently different nucleator, *T*_50_ is uncorrelated to *R* in Fig. S4. This result indicates that experiments with a *τ* lower than approximately 10 min and different *R* can be compared to investigate the effect of *T*_th_ on the width of the nucleation distribution, as we did in the experiments shown in Fig. 1. Ultimately, the effect of the sample mass (*m*_w_) was explored. The range of allowed values for *m*_w_ is upper-limited by the volume of the aluminum pan used for the DSC experiments and lower-limited by the sensibility of the DSC equipment. However, in the range of 10.2–17.5 mg, the sample mass was statistically uncorrelated with both *T*_50_ and *T*_10_-*T*_90_ (Supplementary Fig. S5). The value of *T*_50_ is purely stochastic, whereas the differences in *T*_10_-*T*_90_ can be attributed to the previously discussed parameters (τ and Tth).

## Discussion

A large number of scientific papers in the literature deals with the nucleation behavior of ice. Considering that the nucleation temperatures observed in this work are located well above the homogeneous value (i.e., − 40 °C or below), we can indeed declare that nucleation was heterogeneous and was most likely induced by the contact of the water droplet with the interior surface of the container. One may think that the water droplet movements inside the pan may foster nucleation because of topological defects within the pan surface. However, as reported by Campbell et al. in 2015^28^ and confirmed again in 2017^33^, “*while there is considerable evidence which suggests that topography is a vital factor in nucleation from solution and vapor, there is almost no equivalent support for nucleation from the melt*”^28^. In other words, even though the contact with the metallic surface of the DSC pans does induce heterogeneous nucleation, small droplet movements inside the pan should not be held responsible for the changes in the nucleation temperature distributions herein reported.

Moreover, it is possible to rule out the so-called pre-activation effect. Various materials and particles showed the ability to nucleate ice at higher temperatures compared to their inherent activation temperatures when subjected to repeated nucleation events or when exposed to cold temperatures beforehand^34^. Even though the reported effect may resemble the pre-activation effect, some other considerations exclude this may be true. Indeed, the pre-activation effect was studied for the case of ice nucleation from the vapor and was often correlated to either the incomplete sublimation of ice surface layers on the nucleating particles or the presence of ice in the nanoscopic pores on the particles' surface^34,35^. The experiments showing the pre-activation effect were always performed by maintaining the temperature of the system below 0 °C and modifying the relative humidity to induce nucleation. It is widely reported that the pre-activation disappears when the particles are heated above 0 °C^34^. In our experiments, ice nucleates from the melt, not from the vapor, and the system is heated after every cycle to a temperature of at least 20 °C, meaning that no pre-activation could take place in our experiments.

Eventually, the effect described in this work could be associated with the so-called inside-out contact nucleation^36^. In 2005 Durant^36^ showed that the nucleation temperature of a supercooled water droplet changes drastically depending on the position, either in bulk or at the water–air interface, of an intentionally-introduced ice nucleator. Therefore, the results herein observed for the distribution of nucleation temperatures might be associated with the random movement of an accidentally-introduced nucleator from the bulk to the droplet's surface. Conditions of augmented thermal agitation (higher *τ* or *T*_th_) would correspond to a higher probability of a Brownian movement of the nucleator, jumping between the surface and the bulk of the sample, inducing a high variability in nucleation temperature. In conditions of low thermal agitation, the nucleator would have neither the time nor the energy to change its initial position, and the nucleation temperature distributions observed are correspondingly narrow.

From Einstein’s relationship for the mean square displacement of a particle in a fluid, ${\overline{x} }^{2}$, we get (for a 3D system),

1 $${\overline{x} }^{2}={\int }_{0}^{t}6D(T)dt$$

where *D* is the diffusivity of the particle for which the mean square displacement is being computed, which is generally a function of the temperature *T*, and *t* is the time. The diffusivity *D* is, in turn, related to the viscosity *μ* of the solvent (water in our case) by the Stokes–Einstein relation,

2 $$D=\frac{{k}_{B}T}{6\pi \mu r}$$

where *k*_*B*_ is the Boltzmann constant, and *r* is the size of the particle.

The viscosity of water has been shown to depend on temperature according to the following power-law equation, which was also validated in the supercooled regime^37^,

3 $$\mu ={\mu }_{0}{\left(\frac{T}{{T}_{S}}-1\right)}^{-\gamma }$$

where *µ*_0_ = 1.3788·10^–4^ Pa s, *T*_*S*_ = 225.66 K and *γ* = 1.6438.

Substituting Eqs. (2, 3) into Eq. (1), and collapsing all variables that do not depend on temperature and time into a single constant *C*, we eventually get,

4 $${\overline{x} }^{2}=C{\int }_{0}^{t}\frac{T}{{\left(\frac{T}{{T}_{S}}-1\right)}^{-\gamma }}dt$$

Given that the mean square displacement can be considered as a good indicator of a particle’s ability to move in the water sample, it comes that the probability of a significant movement of the nucleator, i.e., a change in position producing a noticeable difference in nucleation temperature, is directly proportional to the integral in Eq. (4).

**Figure 4:**
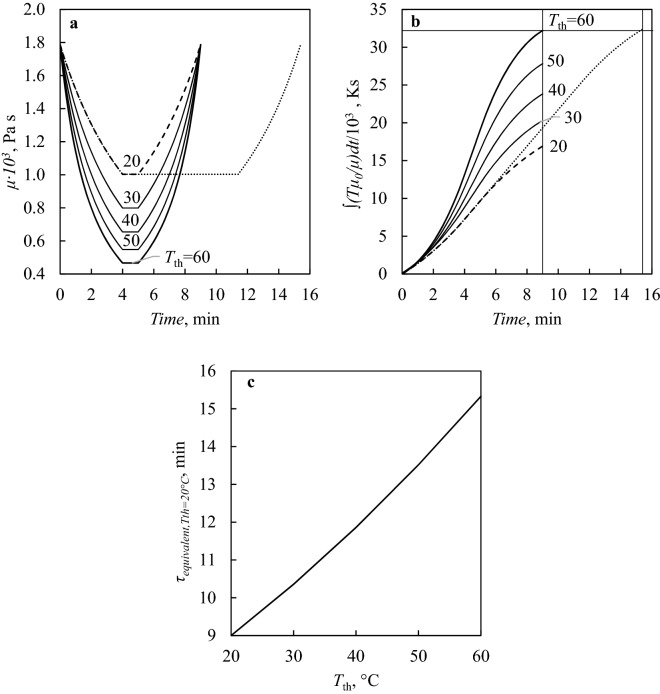
(**a**) Variation of the water viscosity coefficient, *µ*, with time during tests I-V in Table 1, as computed using Eq. (3). (**b**) Running value of the integral in Eq. (4) over time for tests I-V in Table 1: (solid line) run with *T*_th_ = 60 °C and *τ* = 9 min, (dashed line) run with T_th_ = 20 °C and τ = 9 min and (dotted line) hypothetic run with *T*_th_ = 20 °C and *τ* = 15.3 min. The remaining solid lines refer to runs with *T*_th_ = 30–40–50 °C and *τ* = 9 min. (**c**) Equivalent residence time above 0 °C, *τ*_equivalent_, that a freeze–thaw experiment with *T*_th_ = 20 °C should have to be comparable with runs at different *T*_th_ and *τ* = 9 min.

We, therefore, computed such integral for tests I–V in Table 1. These tests were performed with *τ* equal to 9 min and *T*_*th*_ equal to 20, 30, 40, 50, and 60 °C, respectively. To simplify our considerations, we implicitly assumed that the size *r* of the nucleator was similar for all these five experiments. Based on the integral in Eq. (4), the time spent by the system at higher temperatures should be weighted more, and experiments involving long *τ* and low *T*_th_ should be equivalent to experiments with short *τ* and high *T*_th_. Hence, we computed equivalent (i.e., leading to the same overall ${\overline{x} }^{2}$) residence times above 0 °C, *τ*_equivalent_, that a freeze–thaw experiment with *T*_th_ = 20 °C should have to be comparable with runs at *τ* = 9 min and different *T*_th_ of 30, 40, 50, and 60 °C, respectively. The results of this analysis are illustrated in Fig. 4. We obtained that a run with *T*_th_ = 60 °C, *R* = 15 °C/min and *τ* = 9 min can be considered equivalent to a run with *T*_th_ = 20 °C, *R* = 5 °C/min and *τ* = 15.3 min (see Fig. 4b). In line with these calculations, a run with *T*_th_ = 20 °C, *R* = 5 °C/min, and *τ* = 15.3 min should lead to a broad distribution of nucleation temperatures, similar to the distribution obtained at *T*_th_ = 60 °C and *τ* = 9 (test V in Table 1, see Fig. 1). Based on our integrations of the self-diffusion coefficient of water, tests II, III, and IV in Table 1, which were performed at *T*_th_ = 30, 40, and 50 °C, respectively, would correspond to runs carried out at *T*_th_ = 20 °C, *R* = 5 °C/min and *τ* = 10.4, 11.9 and 13.5 min, respectively (Fig. 4c). This result explains why tests with high *τ* in Fig. 3 showed wide nucleation temperature distributions.

Given that it is almost impossible to obtain nucleators-free water samples, it is probable that nucleation was induced by an external particle, rather than solely by the contact between water and the DSC pan. If this is the case, the stochastic Brownian motion of the nucleator could explain the behavior we discussed in this work.

**Figure 5:**
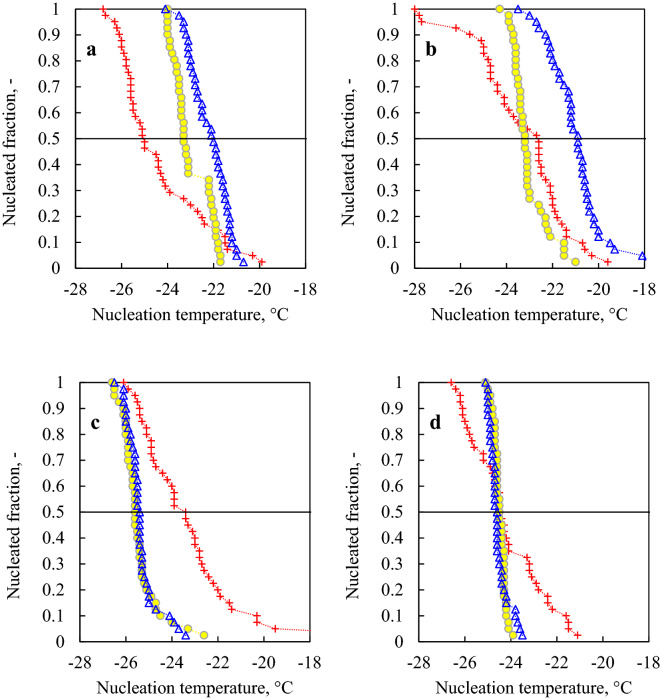
Nucleated fraction vs. nucleation temperature for (**a**) experiment VI, (**b**) experiment VII, (**c**) experiment VIII and (**d**) experiment IX. In all panels: blue triangles, yellow circles, and red crosses correspond respectively to the sub-runs at *T*_th_ = 20 °C, 40 °C, and 60 °C.

In addition, following the classical nucleation theory, one would expect the median nucleation temperature to be correlated with the cooling rate employed in the supercooled region, because the probability of a nucleation event increases with the time spent below the equilibrium melting value. This effect is not appreciable when experiments with different water samples/DSC pans are compared, as the median nucleation temperature depends on the nature of the heterogeneous nucleator present in the sample/pan combination. However, it should be visible in experiments where the same water sample experienced different R values in the supercooling phase (such as experiments VI and VII). As shown in Fig. 5a, the survival curves (and median nucleation temperature values) do translate to lower temperatures as *T*_th_ and*,* hence, *R* increases. In Fig. 5b, however, while there is a clear decrease in median nucleation temperatures between the sub-runs at *T*_th_ = 20 °C and *T*_th_ = 40 °C, the same does not apply to the sub-run at *T*_th_ = 60 °C. Nonetheless, the distribution at *T*_th_ = 60 °C in Fig. 5b has a long tail pointing toward cold nucleation temperatures (*T*_min_ = − 28 °C), in line with the classical nucleation theory. Accordingly, in experiments VIII and IX, Fig. 5c,d, respectively, in which the cooling ramp in the supercooled region did not change, the survival curves of the sub-runs at *T*_th_ = 20 °C and *T*_th_ = 40 °C were virtually identical. Given the low values of *T*_th_ and *τ* employed in these sub-runs, the probability that the nucleator would move and affect the nucleation temperature is extremely low, and the initial position of the nucleator, therefore, dictates the value of T50. Moreover, in both experiments VIII and IX, the sub-runs at *T*_th_ = 60 °C showed a wider distribution of nucleation temperatures, confirming the hypothesis that a more pronounced thermal agitation might induce larger/more frequent movements of the nucleator. It is equally likely that a contact nucleus would move from the bulk to the surface, or vice versa. A nucleus migrating from the surface to the bulk would result in a shift towards lower temperatures of T_90_. Vice versa, a nucleus migrating to the surface would lead to an increment of T_10_. Considering that we do not know in which position the nucleus is at the beginning of the experiments, both situations are possible and equally likely, hence explaining the spread in both directions of the distributions. In this case, the median nucleation temperature is not any more strictly related to the initial position of the nucleator inside the water sample.

## Conclusions

In this work, we have observed a marked effect of thawing temperature and time spent above 0 °C on the heterogeneous nucleation of water. The width of the distribution seems to be directly related to the rate and extent of diffusive processes in water. Conditions of high diffusion translate into broad, scattered distributions, while conditions of low diffusion translate into narrow distributions. This effect could be attributed to two interplaying effects, the inside-out contact nucleation and the Brownian motion of the nucleator within the studied sample. In conditions of high thermal agitation, the Brownian motion induces the nucleator to repeatedly change its position inside the water sample, and the different positions of the nucleator result in nucleation occurring in a wide temperature range.

## Materials and methods

The differential scanning calorimetry (DSC) analyses were carried out using a differential scanning calorimeter (DSC type Q200, TA Instruments, New Castle, DE, USA), equipped with a refrigerated cooling system and a nitrogen line for cell purge (at 50 mL/min), and calibrated with indium. The temperature sampling was performed every 0.2 s with an accuracy of ± 0.1 °C and a precision of 0.05 °C, as declared by the manufacturer. The nucleation temperature was determined by locating the abrupt spike of the DSC heat signal due to the sudden release of heat induced by the nucleation event. In all the analyses, a small amount (*m*_*w*_, see Table 1) of water for injection (Fresenius Kabi, Verona, Italy) was loaded into hermetic aluminum pans and hermetically sealed inside them. Samples were then analyzed against an empty pan as a reference. Further details on the tests performed, including the maximum and minimum temperature values of the cycles (*T*_th_ and *T*_f_, respectively), the cooling/heating rate employed (*R*), the holding time (*t*_h_) at the thawing temperature *T*_th_ for each cycle, the residence time above 0 °C (*τ*) and the total number of cycles performed (*n*_c_) are listed in Table 1.

## Supplementary Information

Supplementary Figures.

Supplementary Information 2.

## Data Availability

All data generated or analyzed during this study are included in this published article. The raw datasets are available in the supplementary information files as an Excel datasheet.
