# Supplementary material for: Effect of diffusion kinetics on the ice nucleation temperature distribution
Source: Sci Rep. 2022 Sep 29;12:16334. doi: 10.1038/s41598-022-20797-1 (PMC9522862; doi:10.1038/s41598-022-20797-1)
Supplement: Supplementary file 1 — Supplementary Figures. [file 41598_2022_20797_MOESM1_ESM.docx]

Supporting Information For

Effect of Diffusion Kinetics on the Ice Nucleation Temperature Distribution

Lorenzo Stratta, Andrea Arsiccio & Roberto Pisano*.

Molecular Engineering Laboratory (molE), Department of Applied Science and Technology, Politecnico di Torino, 24 corso Duca degli Abruzzi, IT-10129 Torino, Italy

Corresponding Author

* Correspondence to: roberto.pisano@polito.it.


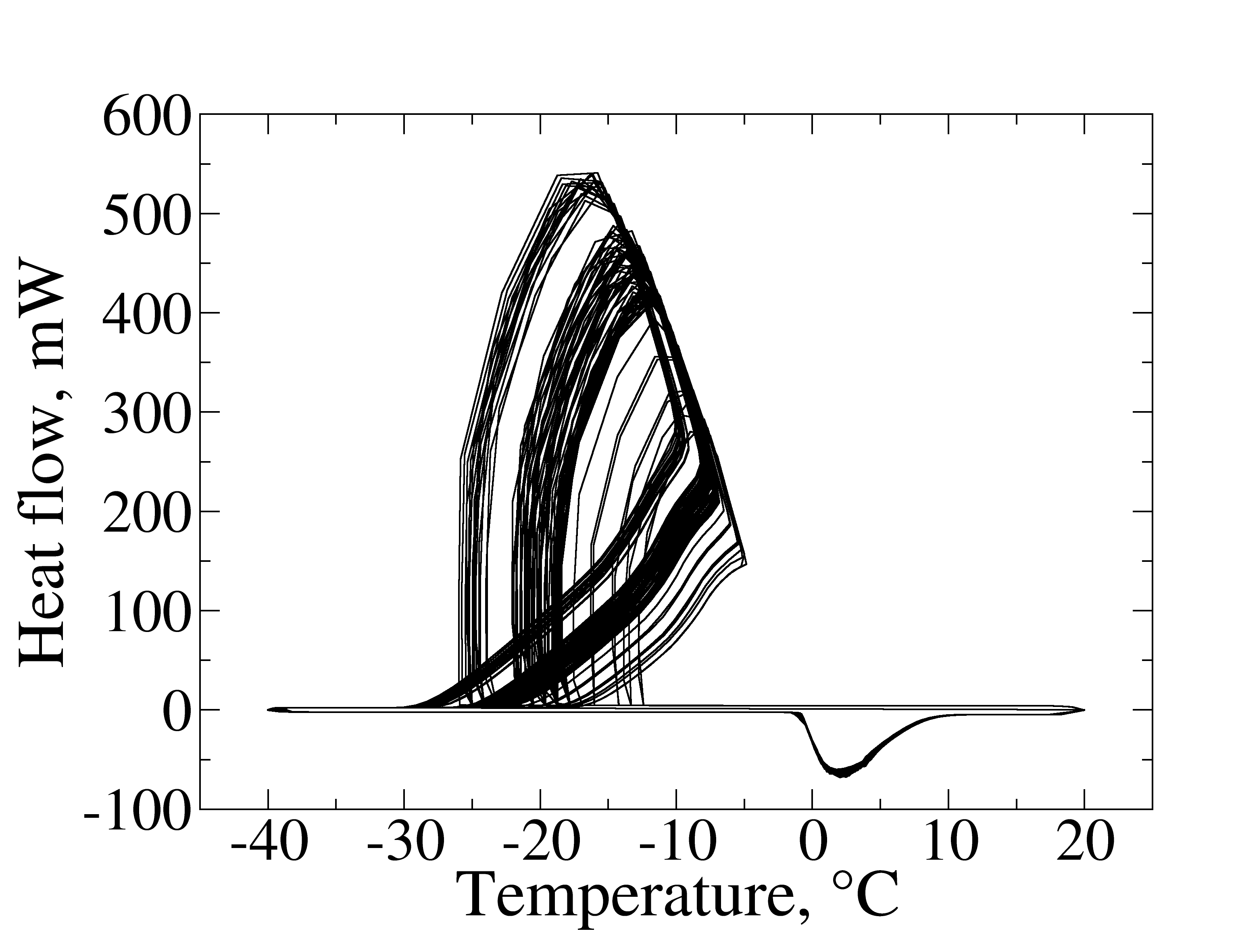


**Figure S1.** Thermogram produced during experiment X, plotted against the temperature of the sample. While the nucleation temperatures are highly scattered, the melting peaks are superimposed.

**Figure S2.** Median nucleation temperature *T*_50_ versus the residence time above 0°C (*τ** *in logarithmic scale experiments I-V, X-XXIV in Table 1). The error bars show the width of the distributions, with the upper and lower values corresponding to *T*_10_ and *T*_90_,respectively. There is no clear correlation between * *and *T*_50_ (the omnibus test fails with a p-value of 0.43, F-value 1.16, degrees of freedom 12).

**Figure S3.** Width of the nucleation temperature distribution (*T*_10_-*T*_90_, in °C) versus the average nucleation temperature *T*_50_ (Experiments I-V, X-XXIV in Table 1). There is no clear correlation between *T*_50_ and *T*_10_-*T*_90_ (the omnibus test fails with a p-value of 0.78, F-value 0.62, degrees of freedom 16).

**Figure S4. a**: Average nucleation temperature *T*_50_ versus cooling/heating rate *R*. The error bars show the width of the distribution, with the upper and lower values corresponding to *T*_10_ and *T*_90_,respectively. **b**: Width of the nucleation temperature distribution (*T*_10_-*T*_90_, in °C) versus cooling/heating rate *R*. Experiments I, and XVI-XXI in Table 1 were used for this analysis.

**Figure S5.** **a**: Average nucleation temperature *T*_50_ and **b**: its distribution *T*_10_-*T*_90_ versus sample mass *m*_w._ The error bars show the width of the distribution, with the upper and lower values corresponding to *T*_10_ and *T*_90_,respectively (Experiments I-V, X-XXIV in Table 1). The omnibus test indicates absence of any significant correlation between *m*_w_ and either *T*_50_ (F-value 1.04, degrees of freedom 14, p-value 0.51) or *T*_10_-*T*_90_ (F-value 0.92, degrees of freedom 14, p-value 0.58).
